# X-ray-charged bright persistent luminescence in NaYF_4_:Ln^3+^@NaYF_4_ nanoparticles for multidimensional optical information storage

**DOI:** 10.1038/s41377-021-00575-w

**Published:** 2021-06-23

**Authors:** Yixi Zhuang, Dunrong Chen, Wenjing Chen, Wenxing Zhang, Xin Su, Renren Deng, Zhongfu An, Hongmin Chen, Rong-Jun Xie

**Affiliations:** 1 State Key Laboratory of Physical Chemistry of Solid Surface, Fujian Provincial Key Laboratory of Materials Genome and College of Materials, Xiamen University, Xiamen, 361005 China; 2 Institute for Composites Science Innovation, School of Materials Science and Engineering, Zhejiang University, Hangzhou, 310027 China; 3 School of Materials Science and Chemical Engineering, Ningbo University, Ningbo, Zhejiang, 315221 China; 4 Key Laboratory of Flexible Electronics (KLOFE) & Institute of Advanced Materials (IAM), Nanjing Tech University, Nanjing, 211800 China; 5 State Key Laboratory of Molecular Vaccinology and Molecular Diagnostics & Center for Molecular Imaging and Translational Medicine, School of Public Health, Xiamen University, Xiamen, 361102 China

**Keywords:** Nanoparticles, Slow light

## Abstract

NaYF_4_:Ln^3+^, due to its outstanding upconversion characteristics, has become one of the most important luminescent nanomaterials in biological imaging, optical information storage, and anticounterfeiting applications. However, the large specific surface area of NaYF_4_:Ln^3+^ nanoparticles generally leads to serious nonradiative transitions, which may greatly hinder the discovery of new optical functionality with promising applications. In this paper, we report that monodispersed nanoscale NaYF_4_:Ln^3+^, unexpectedly, can also be an excellent persistent luminescent (PersL) material. The NaYF_4_:Ln^3+^ nanoparticles with surface-passivated core–shell structures exhibit intense X-ray-charged PersL and narrow-band emissions tunable from 480 to 1060 nm. A mechanism for PersL in NaYF_4_:Ln^3+^ is proposed by means of thermoluminescence measurements and host-referred binding energy (HRBE) scheme, which suggests that some lanthanide ions (such as Tb) may also act as effective electron traps to achieve intense PersL. The uniform and spherical NaYF_4_:Ln^3+^ nanoparticles are dispersible in solvents, thus enabling many applications that are not accessible for traditional PersL phosphors. A new 3-dimensional (2 dimensions of planar space and 1 dimension of wavelength) optical information-storage application is demonstrated by inkjet-printing multicolor PersL nanoparticles. The multicolor persistent luminescence, as an emerging and promising emissive mode in NaYF_4_:Ln^3+^, will provide great opportunities for nanomaterials to be applied to a wider range of fields.

## Abstract (web summary)

Core-shelled nanoparticles NaYF_4_:Ln^3+^@NaYF_4_ with multicolor narrow-band persistent luminescence enable a new multidimensional optical information-storage technology.

## Graphical abstract

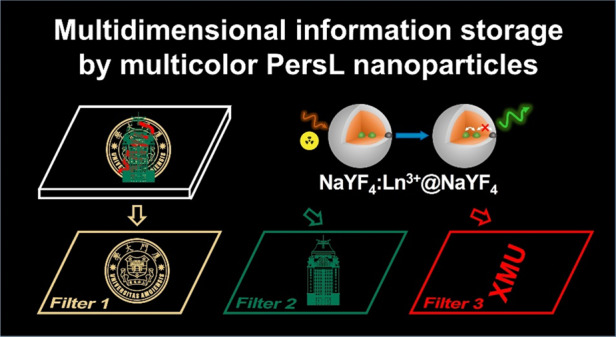

## Introduction

In the past decades, lanthanide-activated NaYF_4_ (NaYF_4_:Ln^3+^) has become one of the best-known luminescent nanomaterials^1–3^. The tremendous and ongoing interest in NaYF_4_:Ln^3+^ mainly comes from its outstanding upconversion characteristics, including the highest upconversion efficiency in single nanoparticles, excellent wavelength tunability via energy-transfer engineering, and superior emission stability against irradiation and heat exposure^4–6^. Consequently, NaYF_4_:Ln^3+^ has found a wide range of applications in in vivo/in vitro bioimaging^7–13^, biological therapy^14–17^, optical sensors^18–20^, 3-dimensional (3D) optical information storage^21,22^, and volumetric 3D displays^23^. Over the past twenty years, the efforts to explore more functionalities in NaYF_4_:Ln^3+^ nanoparticles have never stopped. However, the large specific surface area of nanoparticles easily causes emission quenching, which greatly hinders the discovery and application of new functionalities.

Persistent luminescence (PersL, also known as afterglow) is a slow photon emission resulting from the controlled release of charge carriers from traps in solid-state luminescent materials^24–26^. PersL materials with unique delayed emission have received much attention and exhibit great promise in night-vision security^27–30^, bioimaging^30–42^, optical information storage^43–51^, and anticounterfeiting applications^52,53^. In the biological field, PersL nanoparticles with near-infrared (NIR) emission have been developed as an advanced fluorescent probe for in vivo/in vitro bioimaging^30–42^. NIR PersL imaging technology enables a high signal-to-noise ratio and deep-tissue detection, as clearly demonstrated in small-animal models. Similar to upconversion, the NIR PersL imaging mode can also be merged into multimodal imaging technologies or theranostic platforms, thus opening up new opportunities for future biomedical applications^37,38,40,41^. On the other hand, PersL materials with deep traps capable of storing incident photon energy have been investigated for optical information-storage applications^43,44,46–51^. The readout information delivered by controlled photon emissions includes wavelength and intensity as additional dimensions for each pixel in a plane. This allows wavelength-multiplexing or intensity-multiplexing technologies for multidimensional optical information storage^44,51,54^.

Undoubtedly, whether in bioimaging, optical information storage, or anticounterfeiting, nanoparticles with bright PersL and multicolor emissions are indispensable for practical applications, as the nanoscale size is directly connected to higher spatial resolution and larger information capacity. Although they have long been desired, unfortunately, chemically stable and bright PersL particles with sizes less than 100 nm reported to date are majorly limited to ZnGa_2_O_4_ and its germanium-substituted spinel derivatives. The wavelength of PersL available in these nanoparticles is approximately 700 nm (activated by Cr^3+^) or 500 nm (Mn^2+^).^31–33,35–37,39–41,55,56^ Recently, Han et al. reported multicolor PersL in SiO_2_/CdSiO_3_ hybrid nanoparticles, giving a new direction for high-contrast bioimaging applications.^57^ Also, Ou et al. reported very exciting results of PersL in lanthanide-doped NaLuF_4_:Ln^3+^@NaYF_4_ nanoscintillators to achieve ultralong-lived X-ray trapping for flat-panel-free, high-resolution, three-dimensional imaging^58^. This pioneering work on new PersL nanomaterials opens a window to explore new applications in the information and biological fields.

In this work, multicolor PersL nanoparticles with tunable emission maxima from 480 to 1060 nm are reported in NaYF_4_:Ln^3+^. NaYF_4_ is chosen as the host and produces intense X-ray-irradiated PersL and stable optical performance at a nanoscale. The PersL intensity can be greatly enhanced by using a classical surface-passivated core–shell structure strategy. Excitingly, the lanthanide-doping protocol is effective in realizing PersL color tunability in NaYF_4_:Ln^3+^, enabling a new application in multidimesional optical information storage. The multicolor PersL nanoparticles reported in this work may provide a new emissive mode for the extensively studied NaYF_4_:Ln^3+^ nanomaterials and more possibilities for multifunctional applications. More importantly, this work provides an interesting family of PersL materials with tunable emissions and controllable nanosizes, thus paving an avenue toward optical information storage, anticounterfeiting, and bioimaging applications.

## Results

### PersL in NaYF_4_:Ln^3+^ and the core–shell structure

**Fig. 1:**
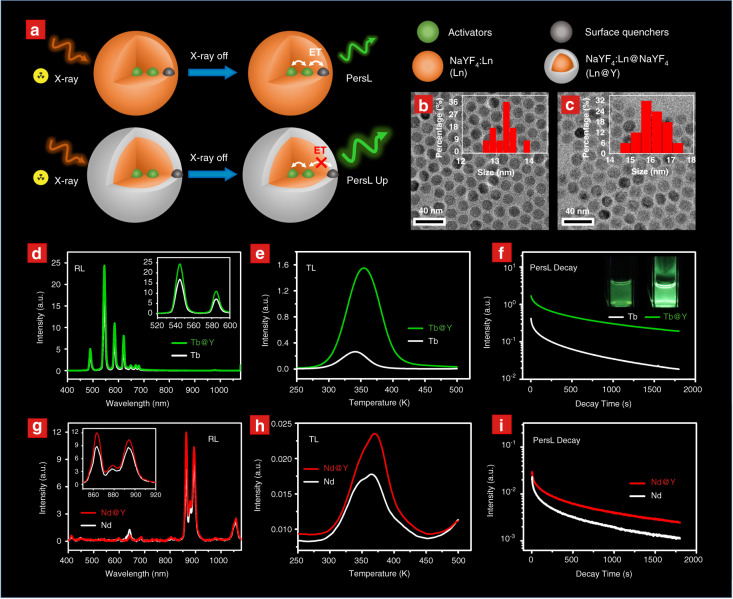
PersL enhancement by the surface-passivated core–shell structure **a** Schematic illustration of the core–shell structure and proposed PersL enhancement mechanism. The description of particle components is given on the right side. **b**–**c** TEM images of NaYF_4_:Tb^3+^ and NaYF_4_:Tb^3+^@NaYF_4_ nanoparticles. The insets show the particle size distributions of the nanoparticles. **d**–**f** RL spectra (**d**), TL glow curves (**e**) and PersL decay curves (**f**) of NaYF_4_:Tb^3+^ and NaYF_4_:Tb^3+^@NaYF_4_ monitored in the range of 400–750 nm. The insert of (**f**) shows the PersL images of NaYF_4_:Tb^3+^ and NaYF_4_:Tb^3+^@NaYF_4_ dispersed in cyclohexane. **g**–**i** RL spectra (**g**), TL glow curves (**h**) and PersL decay curves (**i**) of NaYF_4_:Nd^3+^ and NaYF_4_:Nd^3+^@NaYF_4_ monitored in the range of 400–1000 nm. The RL spectra in (**d**) and (**g**) were measured under X-ray excitation. The TL glow curves in (**e**) and (**h**) as well as the PersL decay curves in (**f**) and (**i**) were recorded after X-ray irradiation for 5 min

A surface-passivated core–shell structure was adopted for the synthesis of nanoparticles (i.e., NaYF_4_:Ln^3+^@NaYF_4_, as schematically illustrated in Fig. 1a), which would impact a positive effect on the PersL performance, as reported in the upconversion studies^9^. Taking the Tb^3+^-doped nanoparticles as an example, the pure hexagonal phase of *β*-NaYF_4_ was confirmed in the NaYF_4_:Tb^3+^ cores. After coating an Ln^3+^-free shell, there was no change in the XRD pattern (Fig. S1). The TEM images show that the synthesized NaYF_4_:Tb^3+^ cores are well-shaped nanospheres with an average diameter of ~13.3 nm (Fig. 1b). The average size of the NaYF_4_:Tb^3+^@NaYF_4_ nanoparticles is increased to ~16.5 nm (Fig. 1c), indicating that Ln^3+^-free NaYF_4_ is epitaxially grown on the shell layer.

Interestingly, bright-green PersL from NaYF_4_:Tb^3+^ nanoparticles in cyclohexane solution can be observed by the naked eye after X-ray irradiation for 10 min (X-ray with a dose rate of ~2.58 μSv/s from a portable X-ray tube). The PersL intensity of the NaYF_4_:Tb^3+^@NaYF_4_ nanoparticles (in powder) was greatly enhanced compared to that of the cores (the inset of Fig. 1f), giving luminance higher than 0.32 mcd/m2 1800 s after turning off the X-ray source (Fig. S2). The NaYF_4_:Tb^3+^@NaYF_4_ nanoparticles still gave strong PersL even at very low irradiation dose (total dose of 38.7 μSv, irradiation time of 15 s) as shown in Fig. S3a. Moreover, the PersL intensity was linearly increased with the increase of irradiation time up to 4500 s (Fig. S3b), which indicated that the nanoparticles possessed a high storage capacity of X-ray-induced charge carriers in traps and the used X-ray dose rate was quite small to reach saturation charging. This left much room to use an X-ray irradiator with a larger irradiation dose rate.

**Fig. 2:**
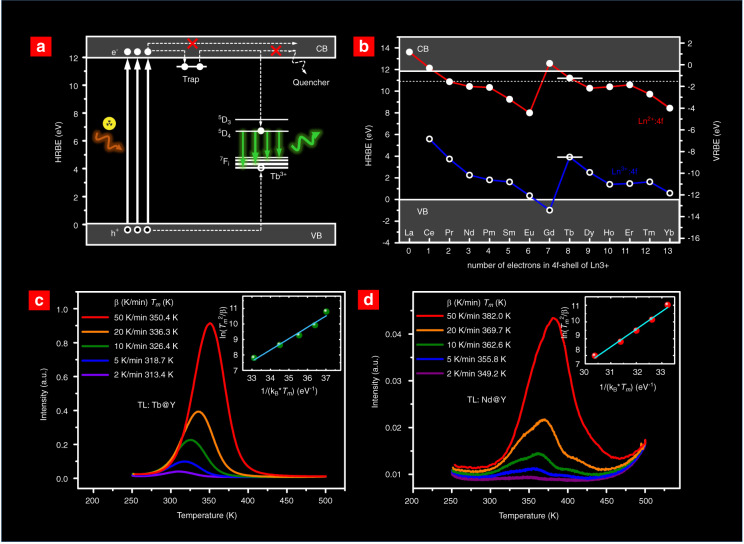
Mechanism of PersL in the NaYF_4_:Ln^3+^ nanoparticles **a** A proposed energy level diagram to interpret the charge carrier transition process in NaYF_4_:Tb^3+^ during and after X-ray irradiation. The energy gap was 11.85 eV, and the GS of Tb^3+^ was 3.92 eV above the VB top according to the data provided by Dorenbos^62^. The trap depth was estimated to be 0.64 eV according to the TL measurements presented in (**c**). The overall process of PersL includes (i) excitation of electron-hole pairs, (ii) capture by electron traps and Tb3+, (iii) thermal release of trapped electrons, and (iv) recombination emission of Tb^3+^. Luminescent quenching routes through electron migration are also depicted, which can be blocked by isolating activators from quenchers. **b** HRBE and VRBE schemes of NaYF_4_. The upper and lower zigzags give the GS of 14 divalent lanthanides (Ln^2+^:4f) and 14 trivalent lanthanides (Ln^3+^:4f). **c**–**d** TL glow curves of NaYF_4_:Tb^3+^@NaYF_4_ (**c**) and NaYF_4_:Nd^3+^@NaYF_4_ (**d**) at different heating rates *β* from 2 to 50 K/min. The insets show the corresponding trap depth fitting using the Hoogenstraaten method

To reveal the possible reason for the PersL enhancement, PL spectra, TL glow curves, and PersL decay curves of the two samples were characterized. As shown in Fig. 1d, the X-ray-excited luminescence (RL) intensity of Tb^3+^ at ~550 nm is enhanced by 1.5 times after the NaYF_4_ coating (Fig. 1d). Additionally, the TL intensity and the PersL intensity of the NaYF_4_:Tb^3+^@NaYF_4_ nanoparticles are both ~5 times higher than those of the cores (Fig. 1e, f). On the other hand, the peak temperature and the range of the two TL glow curves are almost identical, suggesting that the PersL would be originated from the same traps. The above results indicate that the surface passivation strategy that isolates the activators away from surface quenchers is valid to block energy transfer to surface quenchers (Fig. 1a)^59,60^. Moreover, the enhancement of PersL is more significant than that of RL. This is possibly because the charge carrier transferring from the traps to the surface quenchers could also be blocked by the surface passivation process (also refer to the energy-level diagram in Fig. 2a).

We further studied the effect of the core–shell structure on the PersL properties of other NaYF_4_:Ln^3+^ nanoparticles. The RL and PersL intensities in all of the synthesized NaYF_4_:Ln^3+^ nanoparticles are enhanced after growing the NaYF_4_ coating (Ln = Nd in Fig. 1g, i; Ln = Er, Dy, Ho, and Tb@Eu in Figs. S4, S5). Additionally, the emission intensity in the TL glow curve is entirely increased without greatly changing the TL peak temperatures (Ln = Nd in Fig. 1h; Ln = Er, Dy, Ho, and Tb@Eu in Fig. S6). Accordingly, the surface passivation strategy based on the core–shell structure should be a general and effective route to optimize the PersL properties in nanoparticles with a large surface area. In the following sections, the core–shell structure was adopted, unless otherwise specified. TEM images of NaYF_4_:Ln^3+^ and NaYF_4_:Ln^3+^@NaYF_4_ nanoparticles (Ln = Dy, Ho, Nd, Er, and Tb@Eu) are given in Figs. S7–S11.

### Multicolor PersL achieved by lanthanide substitution

**Fig. 3:**
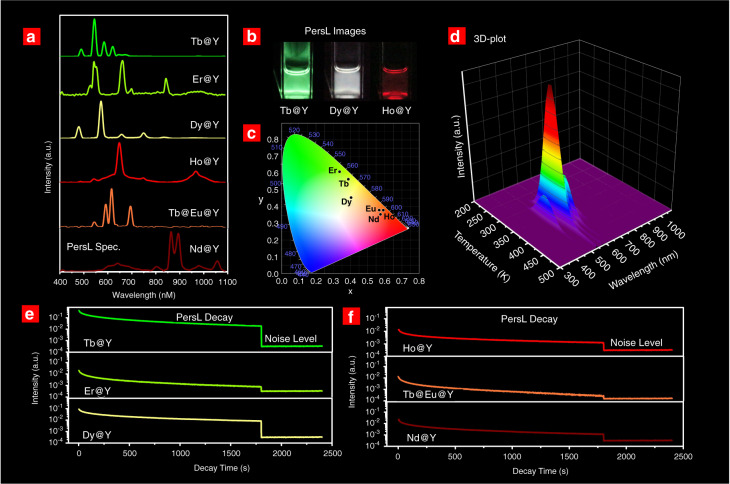
Multicolor PersL in the NaYF_4_:Ln^3+^@NaYF_4_ nanoparticles (Ln = Tb, Er, Dy, Ho, Tb@Eu and Nd) a PersL spectra of thenanoparticles after X-ray irradiation. **b** PersL images of the NaYF_4_:Tb^3+^@NaYF4 (Tb@Y), NaYF_4_:Dy^3+^@NaYF_4_ (Dy@Y) and NaYF_4_:Ho^3+^@NaYF_4_ (Ho@Y) nanoparticles dispersed in cyclohexane. **c** CIE chromaticity diagram of PersL in NaYF_4_:Ln^3+^@NaYF4 nanoparticles. **d** Temperature–wavelength-intensityTL glow graph (3-D plot) for NaYF_4_:Tb^3+^@NaYF_4_. **e**–**f** PersL decay curves in NaYF_4_:Ln^3+^@NaYF_4_ nanoparticles at RT. The nanoparticles wereirradiated by the X-ray source for 10 min before PersL recording. The noiselevel was also given in the curves for comparison. The PersL decay curves ofNaYF_4_:Ln^3+^@NaYF_4_ (Ln = Tb, Er, Dy, Hoand Tb@Eu) monitored in the range of 400–750 nm. The PersL decay curves of NaYF_4_:Nd^3+^@NaYF_4_ monitored in the range of 400–1000 nm

The emission wavelength of PersL is highly significant for optical information storage, anticounterfeiting, and bioimaging applications; however, tuning the PersL wavelength is still a major challenge for nanoparticles. In this work, considering that the Y^3+^ sites in the NaYF_4_ host can provide suitable accommodation for a variety of trivalent lanthanides, the lanthanide substitution was adopted to realize multicolor PersL. As expected, when doping with Tb, Er, Dy, Ho, Tb@Eu, and Nd into the NaYF_4_ cores, multicolor PersL in an ultrawide range from 480 to 1060 nm is achieved (e.g., Dy^3+^: ^4^F_9/2_ → ^6^H_15/2_ at 480 nm; Tb^3+^: ^4^D_4_ → ^7^F_6_ at 490 nm; Er^3+^: ^4^S_3/2_ → ^4^I_15/2_ at 542 nm; **Tb**^**3+**^**:** ^**4**^**D**_**4**_ → ^**7**^**F**_**5**_ **at 545** **nm**; **Dy**^**3+**^**:** ^**4**^**F**_**9/2**_ → ^**6**^**H**_**13/2**_ **at 570** **nm**; Eu^3+^: ^5^D_0_ → ^7^F_2_ at 615 nm; **Ho**^**3+**^**:** ^**5**^**F**_**5**_ → ^**5**^**I**_**8**_ **at 645** **nm**; Er^3+^: ^4^F_9/2_ → ^4^I_15/2_ at 660 nm; Nd^3+^: ^4^F_3/2_ → ^4^I_9/2_ at 862 nm; Nd^3+^: ^4^F_3/2_ → ^6^I_11/2_ at 1060 nm, Fig. 3a). Encouragingly, green (Tb^3+^), white (Dy^3+^), and red (Ho^3+^) PersL of nanoparticles dispersed in cyclohexane can be clearly observed by the naked eye after X-ray excitation at RT (Fig. 3b), which demonstrates their great potentials in inkjet printing and biomedical applications. The emission colors of the synthesized PersL nanoparticles are charted in the Commission Internationale de I’Eclairage (CIE) chromaticity diagram, forming a triangle in the green–white–red area (Fig. 3c).

To determine the origin of multiple PersL bands from a single activator, temperature–wavelength-intensity TL glow graphs (3D plots) were constructed. As shown in Fig. 3d, four narrow TL bands due to the Tb^3+^: ^5^D_4_ → ^7^F_3,4,5,6_ transitions are recorded in the NaYF_4_:Tb^3+^@NaYF_4_ nanoparticles. All the emissions give a main TL peak at ~350 K, indicating that they should be originated from the same traps. The independence of TL glow peaks on different transitions is also found in other Ln^3+^-activated nanoparticles (see Figs. S12–S16). A major advantage of multiple narrow-band PersL emissions is that high-quality optical signals can be obtained by selecting a suitable optical filter to cut off other wavelengths. This will be further discussed in the application section.

All of the synthesized nanoparticles show intense PersL at RT, holding PersL intensities higher than the noise level for 1800 s after turning off the X-ray source (Fig. 3e, f). It should be noted that the NaYF_4_:Tb^3+^@NaYF_4_ nanoparticles have much more intense PersL than other Ln^3+^-activated nanoparticles.

### PersL mechanism in NaYF_4_:Ln^3+^ nanoparticles

To understand the PersL mechanism in NaYF_4_:Ln^3+^ nanoparticles, an energy-level diagram is illustrated in Fig. 2a to interpret the charge-carrier transition process. Upon the X-ray irradiation, many ionized electrons are produced by cascading collisions of hot electrons with atoms in the material^61^. The following low-energy collisions may lead to electron excitation from the valence band (VB) to the conduction band (CB), resulting in the formation of many electron–hole pairs. The generated electrons and holes are subsequently captured by electron traps and activators (e.g., Tb^3+^), respectively. After turning off the X-ray source, the trapped electrons can be thermally released to the CB and further migrate to the excited states of Tb^3+^. The green PersL comes from the recombination of electrons and holes of the Tb^3+^ ions. It should be noted that the electrons excited by X-ray or released from traps to the CB may also freely migrate to luminescent quenchers if the defect density is high enough. Thus, blocking the energy transfer toward surface quenchers should be an effective way to achieve efficient X-ray-irradiated PersL in nanoparticles (Fig. 2a).

Furthermore, the host-referred binding energy (HRBE) and vacuum-referred binding energy (VRBE) schemes of NaYF_4_ were constructed based on the experimental values provided by Dorenbos (Fig. 2b)^62^. The ground-state (GS) energies of all 14 divalent lanthanides (upper zigzag, Ln^2+^:4 f) and 14 trivalent lanthanides (lower zigzag, Ln^3+^:4 f) with respect to the CB and VB can be found in Fig. 2b. According to the HEBE scheme, the GS energy of Tb^3+^ is 3.92 eV above the top of the VB, and Tb^2+^ is 0.64 eV below the bottom of the CB.

On the other hand, the trap depth for the observed green PersL in NaYF_4_:Tb^3+^@NaYF_4_ was estimated by employing the following formula proposed by Hoogenstraaten^63^:

1 $$\frac{{\beta E}}{{{\mathrm{k}}_{\mathrm{B}}\cdot T_m^2}} = s{\mathrm{exp}}\left( {\frac{{ - E}}{{{\mathrm{k}}_{\mathrm{B}}\cdot T_m}}} \right)$$

where *β* (K/s) is the heating rate, *E* (eV) is the trap depth, k_B_ is the Boltzmann constant, *T*_m_ (K) is the peak temperature in the TL glow curves, and s (s^−1^) is the frequency factor (Fig. 2c). The straight line fitting of ln(*T*_m_^2^/*β*) against 1/(k_B_*·T*_m_) suggests that the trap depth *E* in NaYF_4_:Tb^3+^@NaYF_4_ is 0.73 eV, which is close to the energy difference between the GS of Tb^2+^ and the bottom of the CB (0.64 eV). Accordingly, we consider that the main traps contributing to the intense PersL of the NaYF_4_:Tb^3+^@NaYF_4_ nanoparticles are probably due to the Tb^2+^ states. In other words, a portion of the Tb^3+^ ions could be temporarily reduced by the X-ray-generated electrons into a metastable trap state, and the other Tb^3+^ ions act as activators. The dual roles of Tb ions as electron traps and activators consequently enable the intense PersL in the Tb^3+^ singly doped NaYF_4_ nanoparticles.

The TL glow curves of other Ln^3+^-activated core–shell NaYF_4_ nanoparticles are also characterized (Figs. 3d, S17–S20). The NaYF_4_:Nd^3+^@NaYF_4_ nanoparticles show multiple TL bands. The trap-depth estimation of the main peak suggests a value of 1.05 eV (Fig. 2d), basically consistent with the energy difference between the GS of Nd^2+^ and the bottom of the CB (1.43 eV). The other TL bands could be attributed to unknown traps that may intrinsically exist in the NaYF_4_ nanoparticles. The TL glow curves of the NaYF_4_:Er^3+^@NaYF_4_ and NaYF_4_:Ho^3+^@NaYF_4_ nanoparticles indicate that the relatively weak PersL may be mainly derived from intrinsic traps (Figs. S18, S19). The NaYF_4_:Tb^3+^@NaYF_4_:Eu^3+^@NaYF_4_ nanoparticles show a TL glow curve similar to that of NaYF_4_:Tb^3+^@NaYF_4_ (Fig. S20), which supports that Eu^3+^ PersL should be stemmed from Tb^3+^ via PersL energy transfer. It should be acknowledged that the contribution of intrinsic traps or Ln-induced traps to PersL for different activators is unclear yet and deserves deeper investigations in further studies.

### Applications of multicolor persistent luminescent nanoparticles

The NaYF_4_:Ln^3+^@NaYF_4_ nanoparticles exhibit desirable morphology (~20 nm in size and a nearly spherical shape), narrow-band multicolor PersL (wavelength tunable from 480 to 1060 nm), and excellent chemical/dispersion stability in various solvents, which suggests great promise in optical information storage, anticounterfeiting, and bioimaging applications. In this work, we focus on optical information storage, aiming to bring new breakthroughs in multidimensional information storage by utilizing the developed multicolor PersL nanoparticles.

**Fig. 4:**
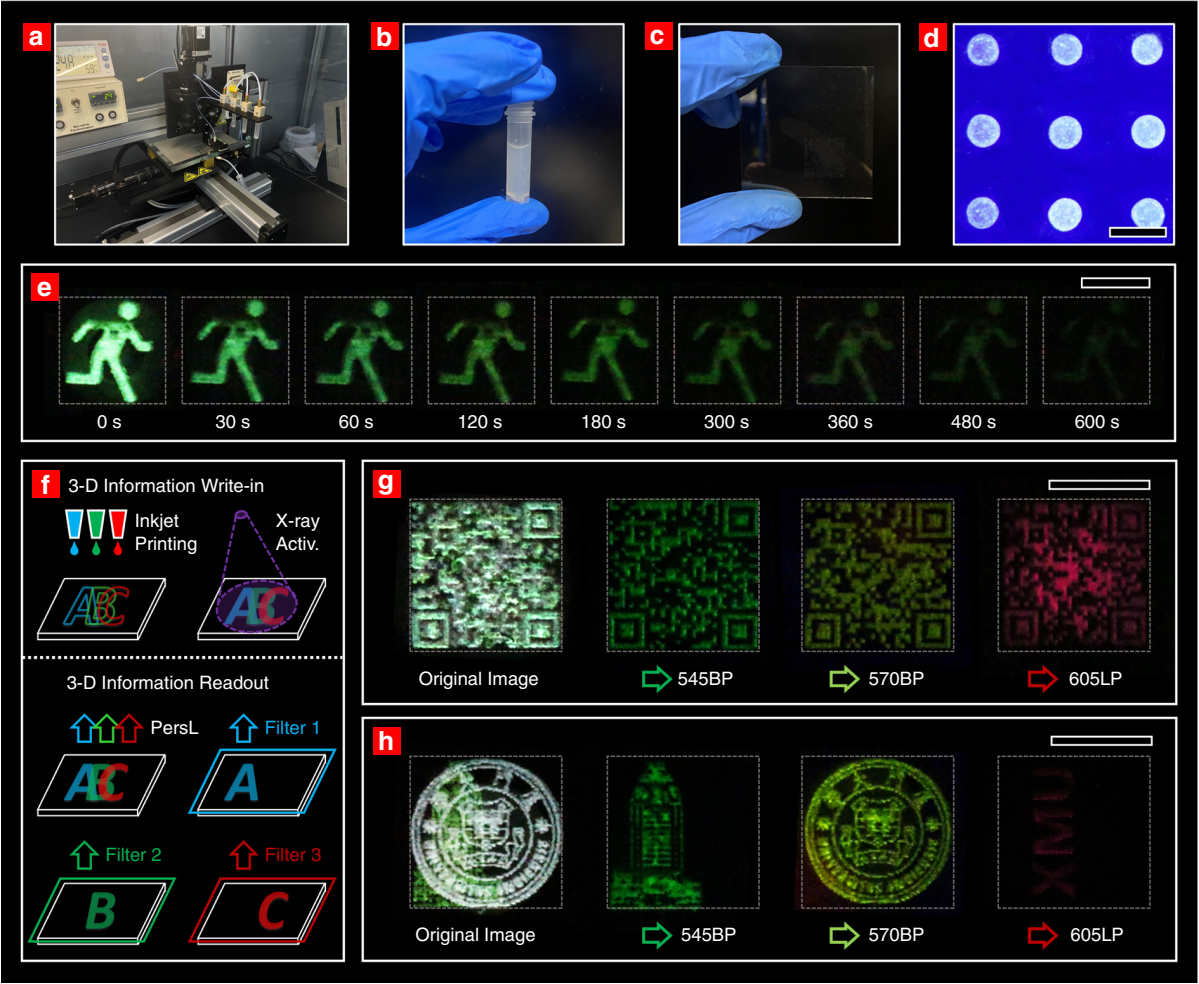
Applications of multicolor persistent luminescent nanoparticles to optical information storage **a**–**c** Photographs of an inkjet printer (**a**), luminescent inks (**b**) and a quartz glass sheet with printed patterns (**c**) under natural light. **d** Photograph of the printed pattern under a fluorescence microscope. The size of a single printed pixel is ~60 μm. **e** PersL images of a printed pattern (emergency exit sign) with delay time from 0 to 600 s. The pattern was printed using the NaYF_4_:Tb^3+^@NaYF4 ink and irradiated by the X-ray source for 5 min. **f** Schematic illustration of the application in 3-D optical information storage. The upper and lower figures illustrate the information write-in and readout processes, respectively. Three independent overlapping patterns were printed on a glass sheet. The luminescent patterns were activated after X-ray irradiation. Mixed information was read directly by an image sensor, and the patterns could be correctly decomposed by using specific optical filters. **g**, **h** The original images and monochromatic images after passing through 545BP, 570BP and 605LP filters. The transmittance spectra of the three optical filters are given in Figs. S21–S23. The scale bars are 100 μm in (**d**) and 10 mm in (**e**, **g**, and **h**), respectively.

An inkjet printing system was applied to output user-defined 2-dimensional (2D) patterns (Fig. 4a). Luminescent inks containing NaYF_4_:Ln^3+^@NaYF_4_ nanoparticles were installed in the printing system (Fig. 4b, photograph of NaYF_4_:Tb^3+^@NaYF_4_ ink). 2D patterns can be printed on a glass sheet, which is hardly recognizable under natural light (Fig. 4c). After X-ray irradiation, the printed 2D pattern made up of long-lasting PersL nanoparticles can be read by an image sensor in the dark. Fig. 4e gives an example of a printed emergency exit sign, which is readable for more than 600 s. The enlarged photograph of the printed pattern under a fluorescence microscope indicates that the size of a single-print dot is ~60 μm (Fig. 4d).

The main principle of a proposed 3D optical information storage scheme is schematically illustrated in Fig. 4f. Three kinds of luminescent inks containing different NaYF_4_:Ln^3+^@NaYF_4_ nanoparticles (Ln = Tb, Dy, and Ho) were applied to print overlapping patterns on the same glass sheet (the information write-in step). Upon X-ray irradiation, the luminescent patterns are activated. Subsequently, the spectral information of each pixel in 2D space was collected by a full-spectral image sensor. The spectral information can be decomposed into three groups of independent patterns after passing through appropriate optical filters (information readout). For example, we printed three quick-response (QR) codes on a glass sheet by using NaYF_4_:Ln^3+^@NaYF_4_ (Ln = Tb, Dy, and Ho) PersL inks. The original luminescent image contains mixed information, which is further interpreted into three readable QR codes after passing through 545BP (exclusively transmittable by the Tb^3+^: ^4^D_4_ → ^7^F_5_ emission), 570BP (Dy^3+^: ^4^F_9/2_ → ^6^H_13/2_), and 605LP (Ho^3+^: ^5^F_5_ → ^5^I_8_) optical filters (Fig. 4g). Additionally, the inkjet-printing scheme based on multicolor PersL nanoparticles can be used to record combined graphic and textual information (Fig. 4h). With these results, the multicolor PersL with narrow emission bands provides several independent wavelength channels for signal readout, thus enabling 3D information storage on a single layer of recording medium. Meanwhile, the signal readout based on PersL shows a lower noise level than the online-excited fluorescence mode, leaving room for achieving a high signal-to-noise ratio. Moreover, these PersL nanoparticles have great potentials for bioimaging applications, as PersL in the first and second biological windows can be realized by doping Nd or Er.

## Discussion

We reported multicolor PersL in NaYF_4_:Ln^3+^@NaYF_4_ nanoparticles with a surface-passivated core–shell structure. The surface-passivation strategy was proven valid to enhance the RL and PersL intensities for all the synthesized nanoparticles by isolating the activators away from the surface quenchers. The NaYF_4_:Ln^3+^@NaYF_4_ nanoparticles showed narrow-band PersL, which was tunable in a broad range from 480 to 1060 nm. By means of TL measurement and the HRBE scheme, a possible mechanism for PersL in NaYF_4_:Ln^3+^ was proposed, which suggested that some lanthanide ions (such as Tb) might act as effective electron traps to achieve intense PersL. We also demonstrated a 3D optical information-storage scheme by using multicolor PersL nanoparticles. We firmly believe that the discovery of multicolor PersL in the NaYF_4_:Ln^3+^ nanoparticles well known for upconversion studies will provide great opportunities for nanomaterials in the fields of optical information storage, anticounterfeiting, and bioimaging.

## Materials and methods

### Chemicals and materials

Tb(CH_3_COO)_3_·6H_2_O (99.9%), Er(CH_3_COO)_3_·6H_2_O (99.9%), Dy(CH_3_COO)_3_·6H_2_O (99.9%), Nd(CH_3_COO)_3_·6H_2_O (99.9%), Ho(CH_3_COO)_3_·6H_2_O (99.9%), Eu(CH_3_COO)_3_·6H_2_O (99.9%), Y(CH_3_COO)_3_·6H_2_O (99.9%), NaOH (99.9%), NH_4_F (99.9%), 1-octadecene (ODE, 90%), and oleic acid (OA, 90%) were used as raw materials for the synthesis of fluoride nanoparticles. Deionized water (prepared in the lab), ethanol (99.7%), cyclohexane (99.7%), acetone (99.5%), or HCl (36%) were used as solvents.

### Synthesis of NaYF_4_:Ln^3+^ nanoparticles

NaYF_4_:Ln^3+^ nanoparticles (cores) were synthesized using a reported coprecipitation method with some modifications^64^. In a typical synthesis procedure, OA (3 mL) and ODE (7 mL) were put into a 50-mL flask. The mixture was added to a water solution (2 mL) containing Y(CH_3_COO)_3_ and Ln(CH_3_COO)_3_ (Ln = Tb, Er, Dy, Ho, Eu, or Nd) in a total amount of 0.4 mmol. The molar ratio of Y/Ln was varied from 95/5 to 80/20. The mixture was heated to 150°C and kept for 1 h to form a transparent colorless solution. After cooling to 50°C, a methanol solution (6 mL) containing NaOH (1 mmol) and NH_4_F (1.6 mmol) was added into the flask and stirred for 30 min. The solution was heated again to 110°C for 30 min to remove volatile components and further to 300°C for 1.5 h under an argon flow to promote the coprecipitation reaction. After cooling to room temperature (RT), the NaYF_4_:Ln^3+^ nanoparticles were collected by centrifugation after adding excessive ethanol into the reaction system. The NaYF_4_:Ln^3+^ nanoparticles were washed with a mixture of ethanol and cyclohexane and dispersed in cyclohexane (4 mL) for further use. For the doping of Eu^3+^, a double-layered structure, NaYF_4_:Tb^3+^@NaYF_4_:Eu^3+^, was adopted to obtain better PersL of Eu^3+^ through the Tb^3+^-to-Eu^3+^ transfer energy. The preparation method was similar to the following synthesis process of NaYF_4_:Ln^3+^@NaYF_4_ core–shell nanoparticles. The optimal doping concentrations for Tb, Dy, Nd, Er, and Ho in the NaYF_4_ nanoparticles are 20, 5, 5, 15, and 15 mol%, respectively, according to the TL glow curve measurements (Fig. S24). The optimal doping concentration of NaYF_4_:Tb^3+^@NaYF_4_:Eu^3+^@NaYF_4_ is NaYF_4_:Tb^3+^(70 mol%)@NaYF_4_:Eu^3+^(30 mol%)@NaYF_4_. The optimal doping concentrations were used for sample preparation unless otherwise specified.

### Synthesis of NaYF_4_:Ln^3+^@NaYF_4_ core–shell nanoparticles

NaYF_4_:Ln^3+^@NaYF_4_ core–shell nanoparticles were prepared by using the obtained NaYF_4_:Ln^3+^ nanoparticles as seeds. The detailed procedure was similar to that the synthesis of cores. Briefly, the same transparent water solution (2 mL) containing OA (3 mL), ODE (7 mL), and Y(CH_3_COO)_3_ (0.4 mmol) was prepared. A cyclohexane dispersion (4 mL) of NaYF_4_:Ln^3+^ nanoparticles (~0.25 mmol) was added to the reaction system along with a methanol solution (6 mL) containing NaOH (1 mmol) and NH_4_F (1.6 mmol). The NaYF_4_:Ln^3+^@NaYF_4_ nanoparticles were then produced using the same reaction conditions and washing method. The NaYF_4_:Ln^3+^@NaYF_4_ nanoparticles were dispersed in cyclohexane (4 mL) for further use.

### Structural and optical characterization

X-ray diffraction (XRD) patterns of the nanoparticles were examined using an X-ray diffractometer (Bruker, D8 Advance) with Cu Kα radiation. The microstructure was observed using a field-emission transmission electron microscope (FE-TEM, FEI, Talos F200s). RL and PersL spectra were recorded at RT with a fiber-type spectrometer (Ocean Optics, QE Pro). The excitation source came from a portable X-ray tube (Amptek, Mini-X2) with a maximum output of 10 W (typical voltage 50 kV, tube current 200 μA, and average dose rate 2.58 μSv/s). The measurement setup for the PersL decay curves and thermoluminescence (TL) glow curves was similar to that reported in our previous work^65^, except that the X-ray tube was used as the excitation source (see Figs. S25, S26). For the measurement of TL glow curves, the nanoparticles (powders) were first exposed to the X-ray source for 60 s at RT. After turning off the excitation, the TL signals were simultaneously monitored with a photomultiplier tube (PMT) detector (Hamamatsu, R928P) and a spectrometer (Ocean Optics, QE Pro). The temperature was controlled by a cooling/heating stage (Linkam, THMS600E), typically from −23°C to 227°C with a rate of 20°C/min. The measurement system was driven by a customer-built LabVIEW operation program. Photographs of PersL-emitting samples (aqueous solutions containing the nanoparticles or inkjet-printed patterns on glass sheets) were taken with a digital camera (Canon, EOS 5D Mark II) in an all-manual mode (exposure time: 3.2 s, aperture: f/3.5, and sensitivity: ISO 12800).

### Preparation of luminescent inks

The NaYF_4_:Ln^3+^@NaYF_4_ (Ln = Tb, Dy and Ho) nanoparticles were centrifuged from cyclohexane and dispersed in acetone (6 mL) after sonicating for 20 min. HCl (0.5 mL) was added to the new solution to remove the surface oleate capping ligands^66^. The ligand-free nanoparticles were collected by centrifugation and redispersed in water to form a stable aqueous ink.

### Applications in optical information storage

An inkjet-printing system for nanomaterial deposition (MicroFab, Jetlab@4) with three different luminescent inks was used to prepare multilayered patterns of nanoparticles. The luminescent inks were loaded into different cartridges connected with 50-μm-diameter piezoelectric-type nozzles. The driving voltage waveforms and negative-pressure values of the inkjet printer were adjusted to generate stable and continuous droplets. The spatial accuracy of the inkjet printing system was ~5 μm. Quartz glass sheets (50 mm × 50 mm × 1 mm) were placed on a substrate under the printing nozzle. The temperature of the substrate was set to 50°C. The entire inkjet-printing process was accomplished in air with ambient humidity higher than 60%. Three overlapping layers of nanoparticle patterns using different luminescent inks were deposited (inkjet-printed) on the quartz glass sheet. The printed patterns were irradiated by the X-ray source for 10 min. The PersL images were acquired by a digital camera, with or without specific optical filters.

## Supplementary information

Supplementary Information
