# Supplementary material for: X-ray-charged bright persistent luminescence in NaYF4:Ln3+@NaYF4 nanoparticles for multidimensional optical information storage
Source: Light Sci Appl. 2021 Jun 23;10:132. doi: 10.1038/s41377-021-00575-w (PMC8222364; doi:10.1038/s41377-021-00575-w)
Supplement: Supplementary file 1 — Supplementary Information [file 41377_2021_575_MOESM1_ESM.docx]

Supplementary Information for

X-ray-charged bright persistent luminescence in NaYF_4_:Ln^3+^@NaYF_4_ nanoparticles for multidimensional optical information storage

Yixi Zhuang^1,^*, Dunrong Chen^1^, Wenjing Chen^1^, Wenxing Zhang^2^, Xin Su^2,3^, Renren Deng^2^, Zhongfu An^4^, Hongmin Chen^5^ and Rong-Jun Xie^1,^*

^1^State Key Laboratory of Physical Chemistry of Solid Surface, Fujian Provincial Key Laboratory of Materials Genome and College of Materials, Xiamen University, Xiamen 361005, China

^2^Institute for Composites Science Innovation, School of Materials Science and Engineering, Zhejiang University, Hangzhou 310027, China

^3^School of Materials Science and Chemical Engineering, Ningbo University, Ningbo, Zhejiang 315221, China

^4^Key Laboratory of Flexible Electronics (KLOFE) & Institute of Advanced Materials (IAM), Nanjing Tech University, Nanjing 211800, China

^5^State Key Laboratory of Molecular Vaccinology and Molecular Diagnostics & Center for Molecular Imaging and Translational Medicine, School of Public Health, Xiamen University, Xiamen 361102, China

*Correspondence: zhuangyixi@xmu.edu.cn; rjxie@xmu.edu.cn


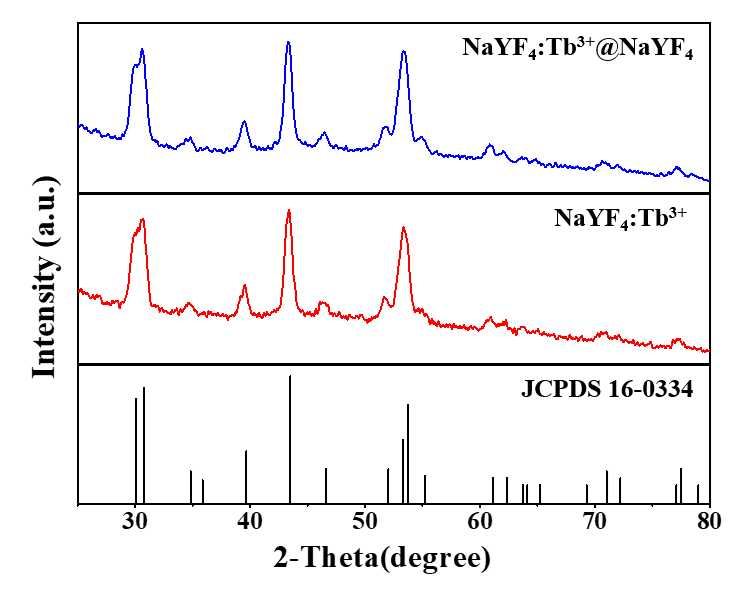


Figure S**1**. XRD patterns of the synthesized NaYF4:Tb^3+^ cores and NaYF4:Tb^3+^@NaYF4 nanoparticles. The bottom gives the standard JCPDS card of the hexagonal NaYF4 crystal (Joint Committee Powder Diffraction Standards #16-0334).


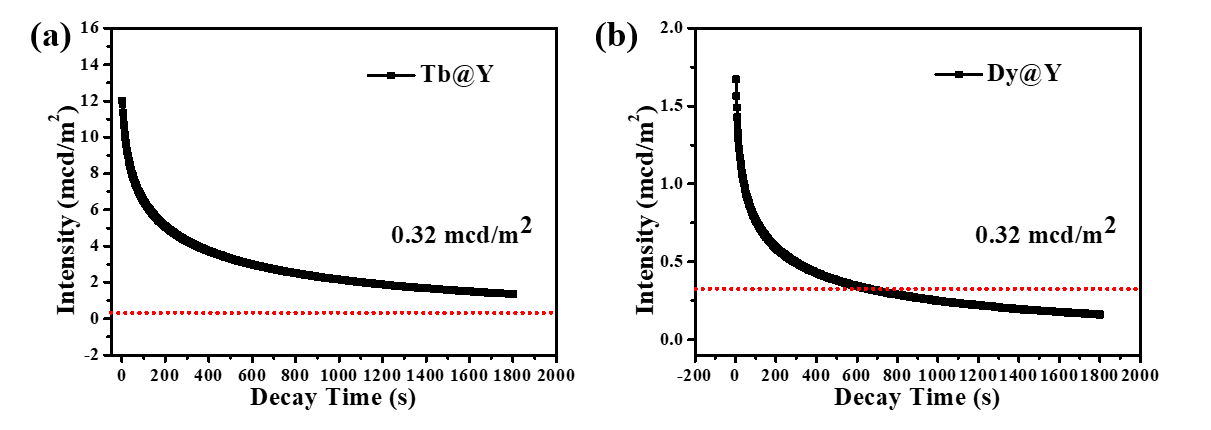


**Figure S2.** PersL decay curves of NaYF_4_:Tb^3+^@NaYF_4_ (a) and NaYF_4_:Dy^3+^@NaYF_4_ nanoparticles (b) monitored in the range of 400-750 nm after irradiated by X-ray for 5 min. The intensity was calibrated by using a luminance meter (Evenfine, LM-5). The straight line of 0.32 mcd/m^2^ gives roughly 100 times of the low limit of human eye sensitivity in dark-adapted condition.


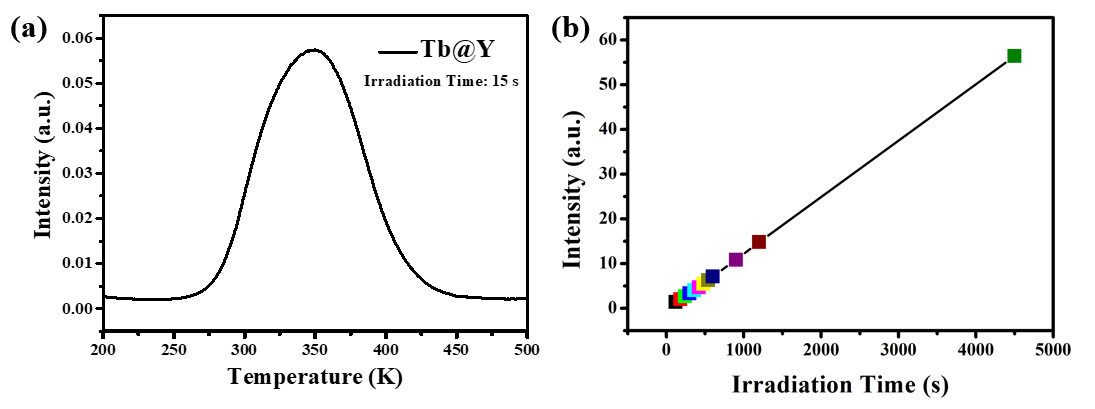


Figure S**3**. (a) TL glow curves of NaYF_4_:Tb^3+^@NaYF_4_ nanoparticles after irradiated by X-ray for 15 s; (b) The linear relationship between the PersL intensity and irradiation time.


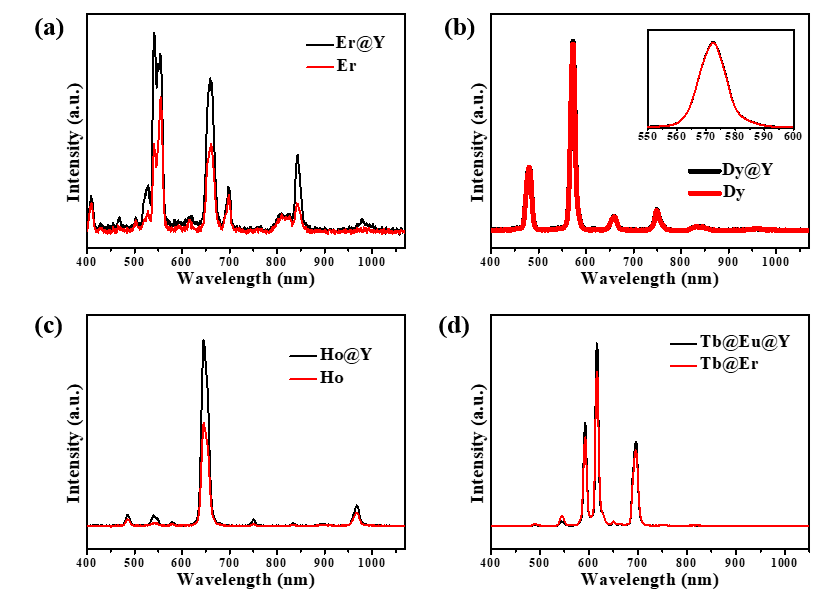


Figure S**4**. RL spectra of NaYF_4_:Ln^3+^ and NaYF_4_:Ln^3+^@NaYF_4_ nanoparticles under the excitation of X-ray source (Ln = Er in (a), Dy in (b), Ho in (c) and Tb@Eu in (d)).


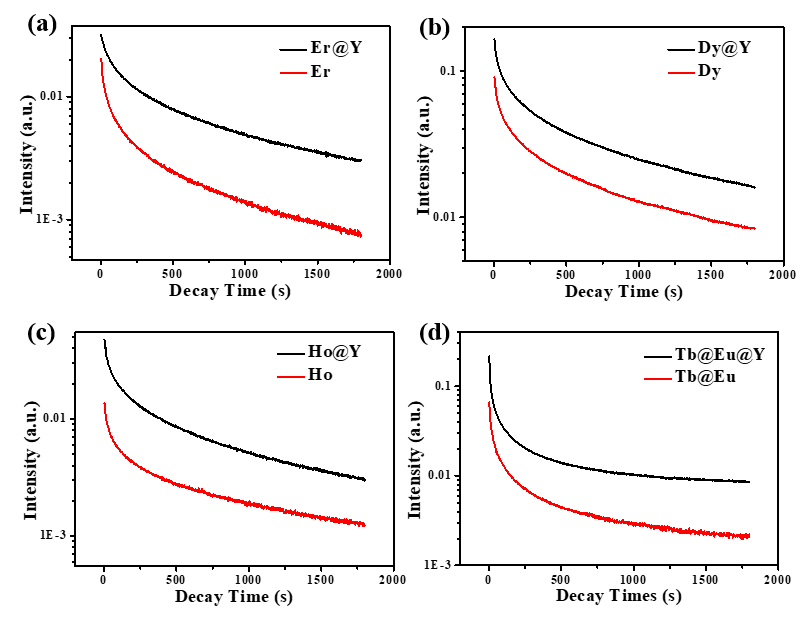


Figure S**5**. PersL decay curves of NaYF_4_:Ln^3+^ and NaYF_4_:Ln^3+^@NaYF_4_ nanoparticles monitored in the range of 400-750 nm after irradiated by X-ray for 5 min (Ln = Er in (a), Dy in (b), Ho in (c), and Tb@Eu in (d)).


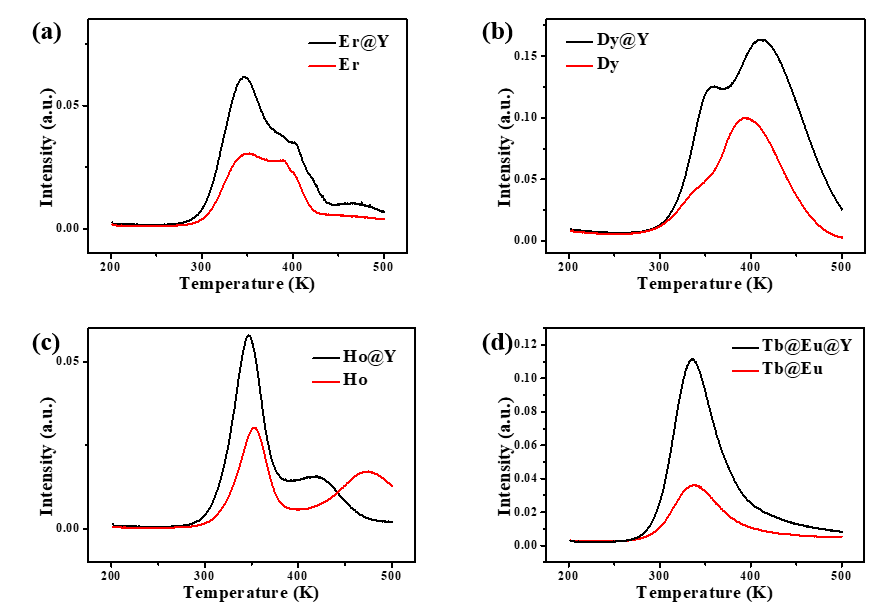


Figure S**6**. TL glow curves of NaYF_4_:Ln^3+^ and NaYF_4_:Ln^3+^@NaYF_4_ nanoparticles after irradiated by X-ray for 5 min (Ln = Er in (a), Dy in (b), Ho in (c) and Tb@Eu in (d)).


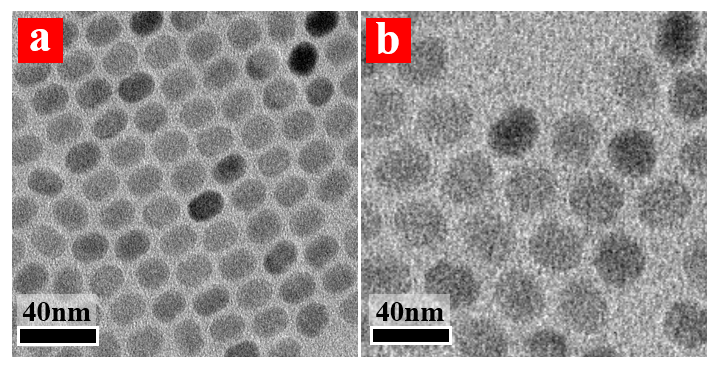


Figure S**7**. TEM images of NaYF_4_:Dy^3+^ (a) and NaYF_4_:Dy^3+^@NaYF_4_ (b) nanoparticles.


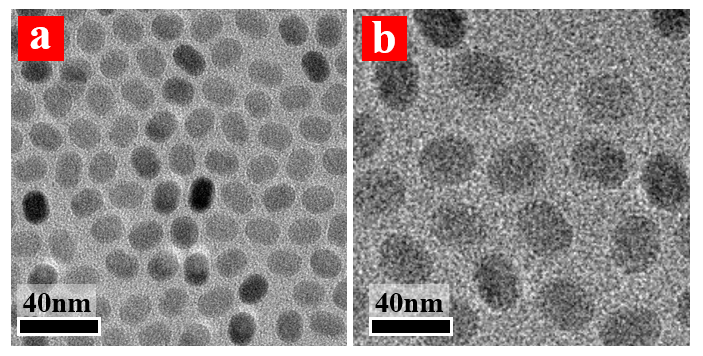


Figure S**8**. TEM images of NaYF_4_:Ho^3+^ (a) and NaYF_4_:Ho^3+^@NaYF_4_ (b) nanoparticles.


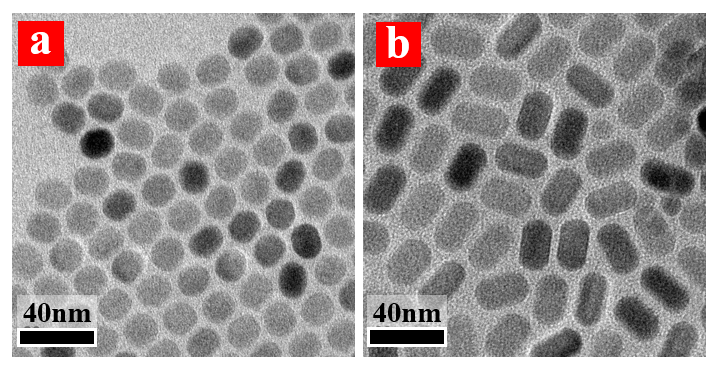


Figure S**9**. TEM images of NaYF_4_:Nd^3+^ (a) and NaYF_4_:Nd^3+^@NaYF_4_ (b) nanoparticles.


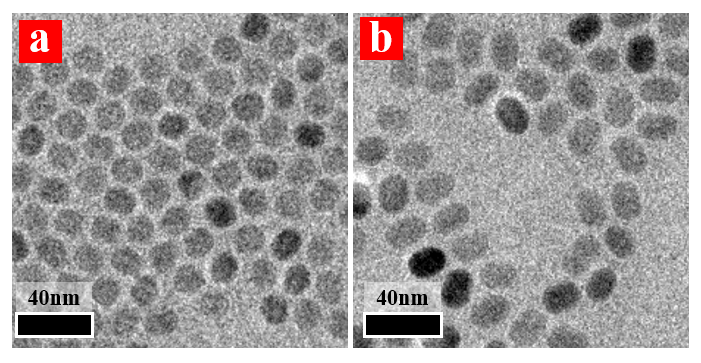


Figure S**10**. TEM images of NaYF_4_:Er^3+^ (a) and NaYF_4_:Er^3+^@NaYF_4_ (b) nanoparticles.


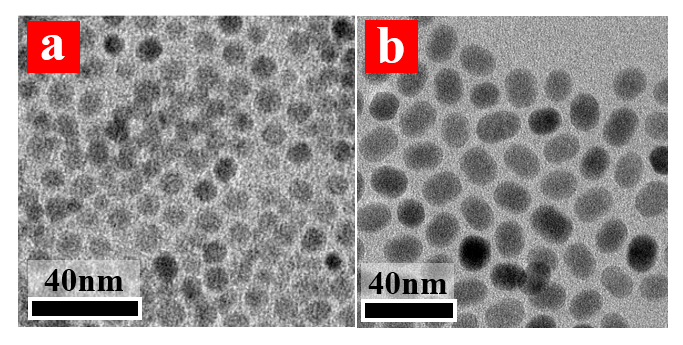


Figure S**11**. TEM images of NaYF_4_:Tb^3+^@NaYF_4_:Eu^3+^ (a) and NaYF_4_:Tb^3+^@NaYF_4_:Eu^3+^ @NaYF_4_ (b) nanoparticles.


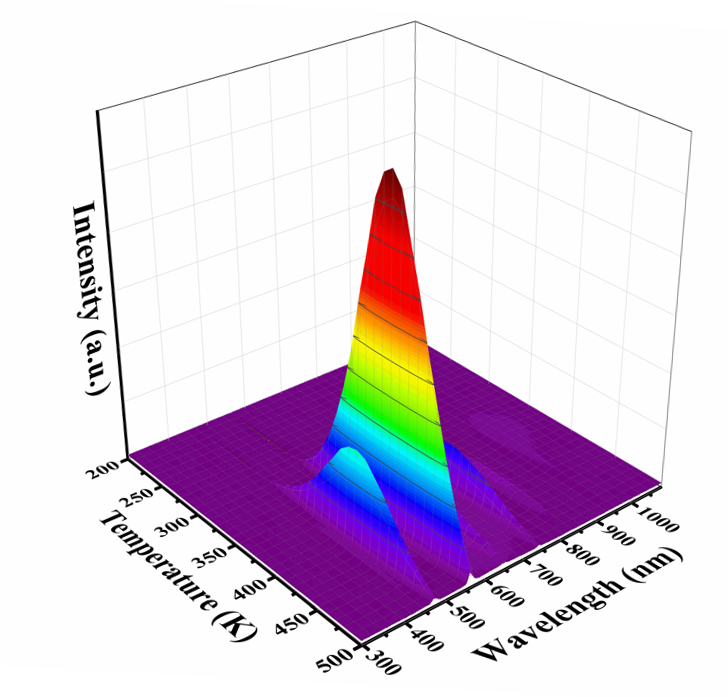


Figure S**12**. Temperature-wavelength-intensity (3D) plot of the PersL in NaYF_4_:Dy^3+^@NaYF_4_.


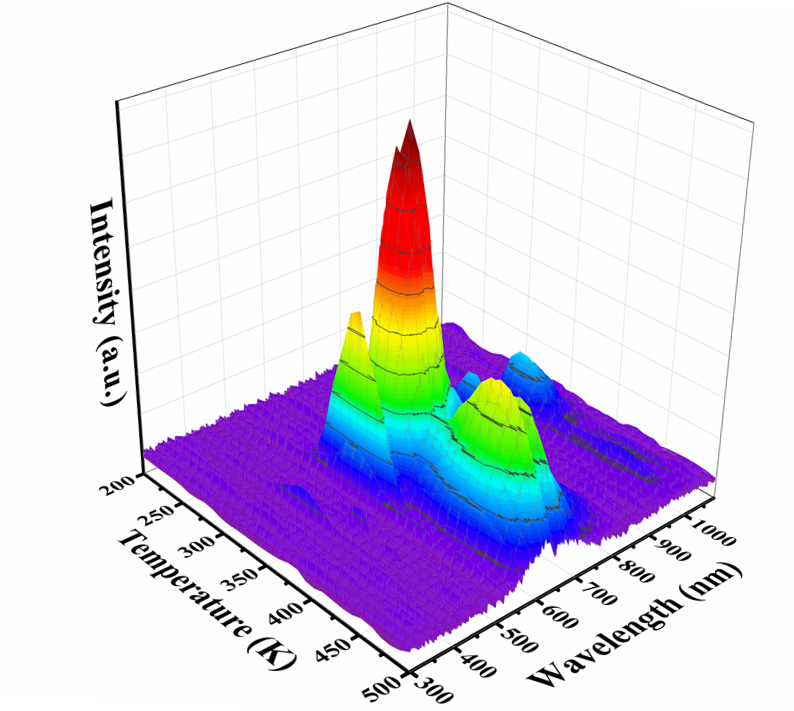


Figure S**13**. Temperature-wavelength-intensity (3D) plot of the PersL in NaYF_4_:Er^3+^@NaYF_4_.


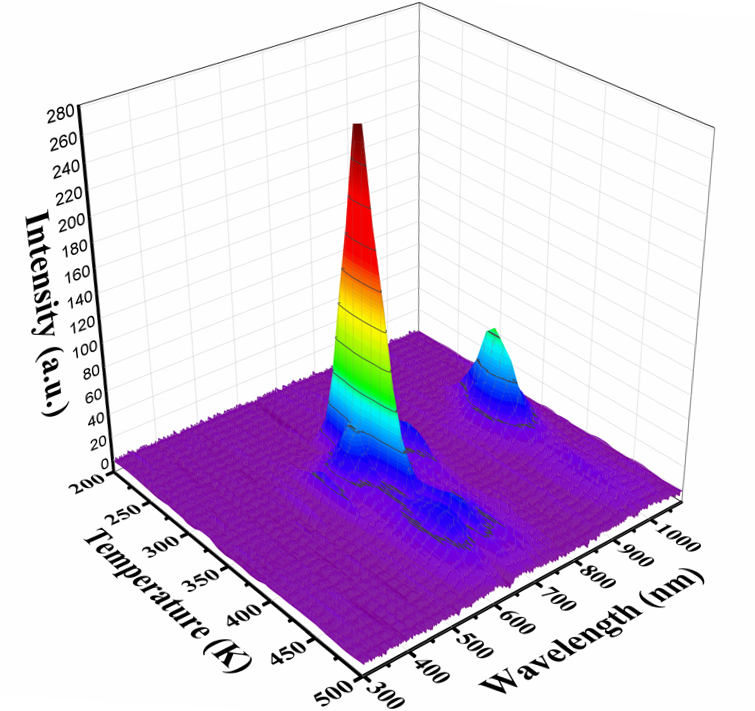


Figure S**14**. Temperature-wavelength-intensity (3D) plot of the PersL in NaYF_4_:Ho^3+^@NaYF_4_.


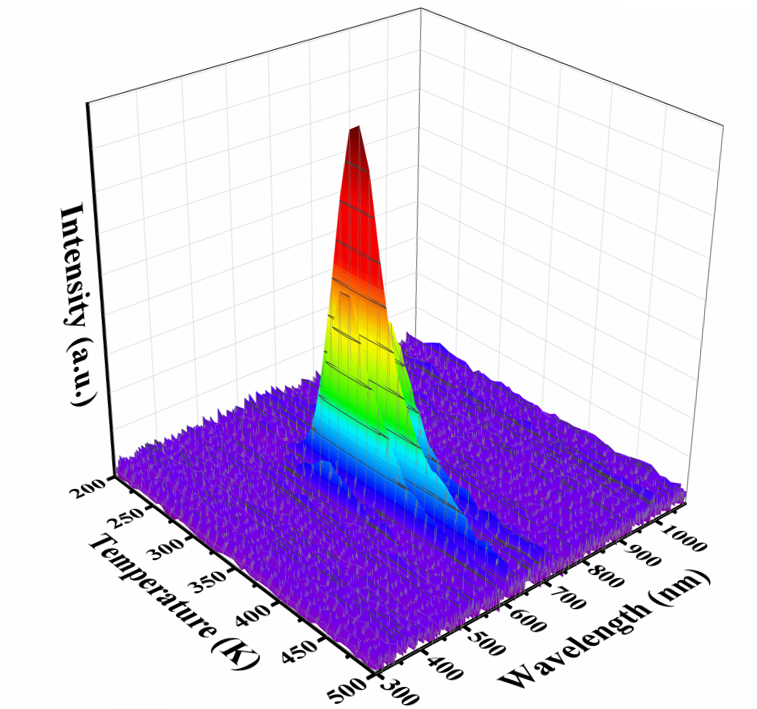


Figure S**15**. Temperature-wavelength-intensity (3D) plot of the PersL in NaYF_4_:Tb^3+^@NaYF_4_:Eu^3+^@NaYF_4_.


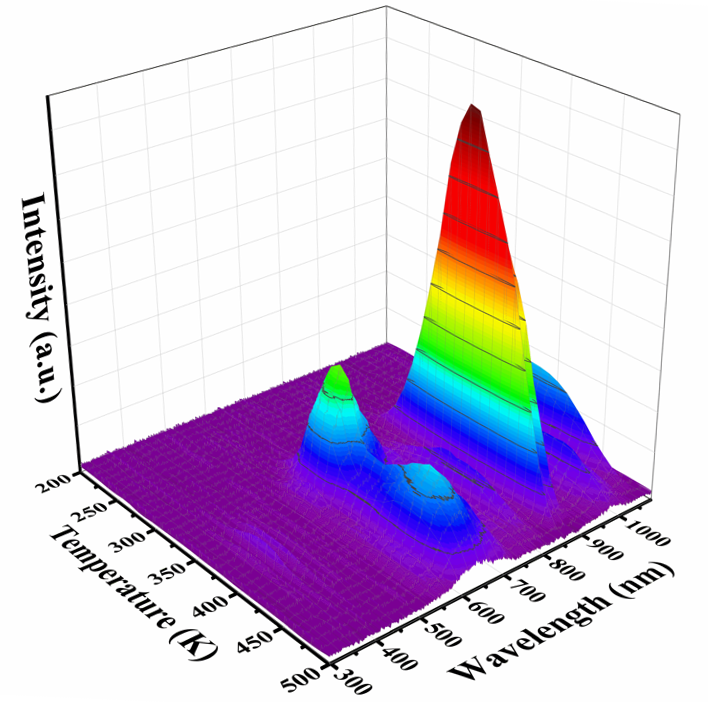


Figure S**16**. Temperature-wavelength-intensity (3D) plot of the PersL in NaYF_4_:Nd^3+^@NaYF_4_.


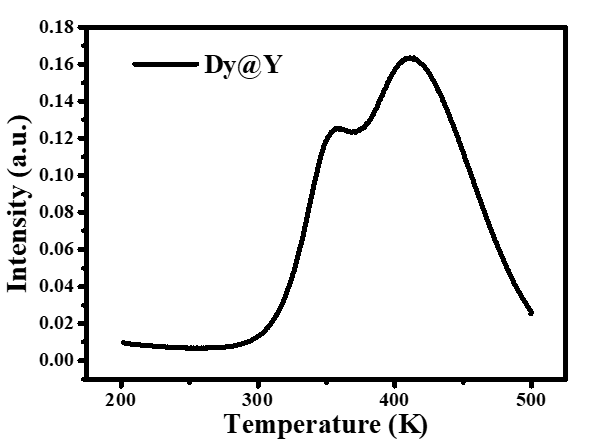


Figure S**17**. TL glow curves of NaYF_4_:Dy^3+^@NaYF_4_ nanoparticles after irradiated by X-ray for 5 min.


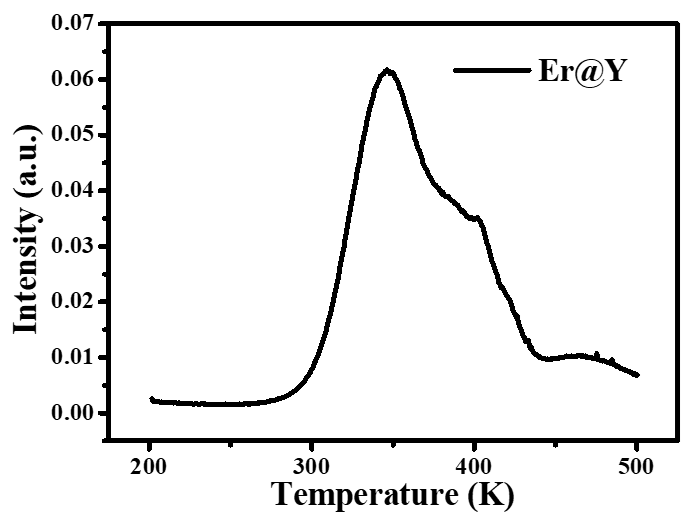


Figure S**18**. TL glow curves of NaYF_4_:Er^3+^@NaYF_4_ nanoparticles after irradiated by X-ray for 5 min.


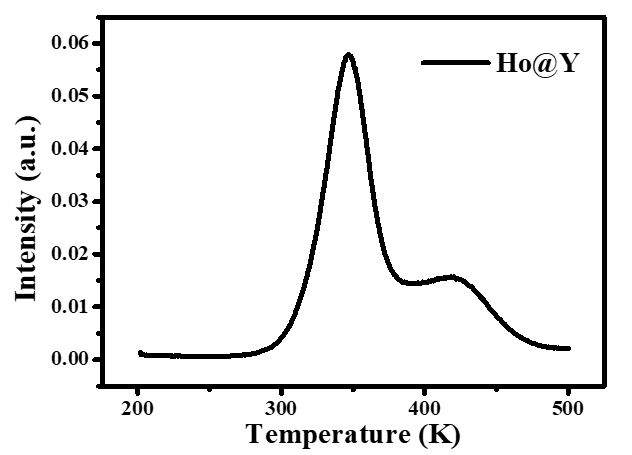


Figure S**19**. TL glow curves of NaYF_4_:Ho^3+^@NaYF_4_ nanoparticles after irradiated by X-ray for 5 min.


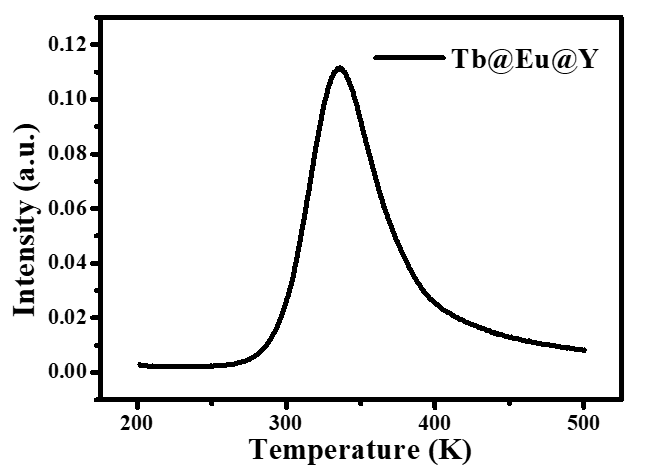


Figure S**20**. TL glow curves of NaYF_4_:Tb^3+^@NaYF_4_:Eu^3+^@NaYF_4_ nanoparticles after irradiated by X-ray for 5 min.


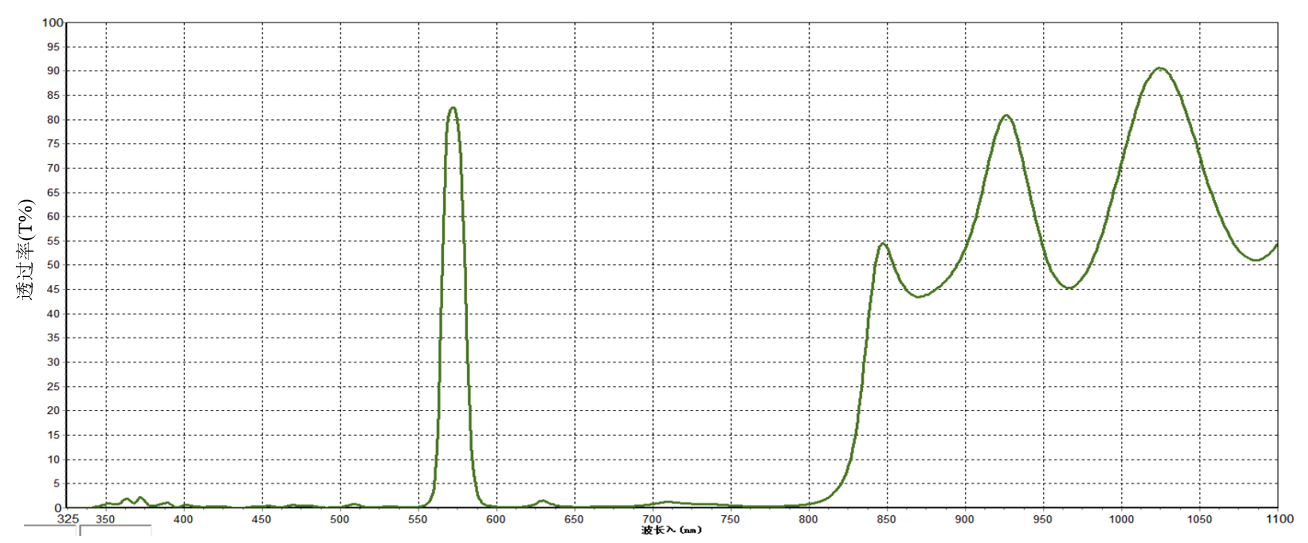


Figure S**21**. Transmittance spectra of band-pass optical filter BP545.


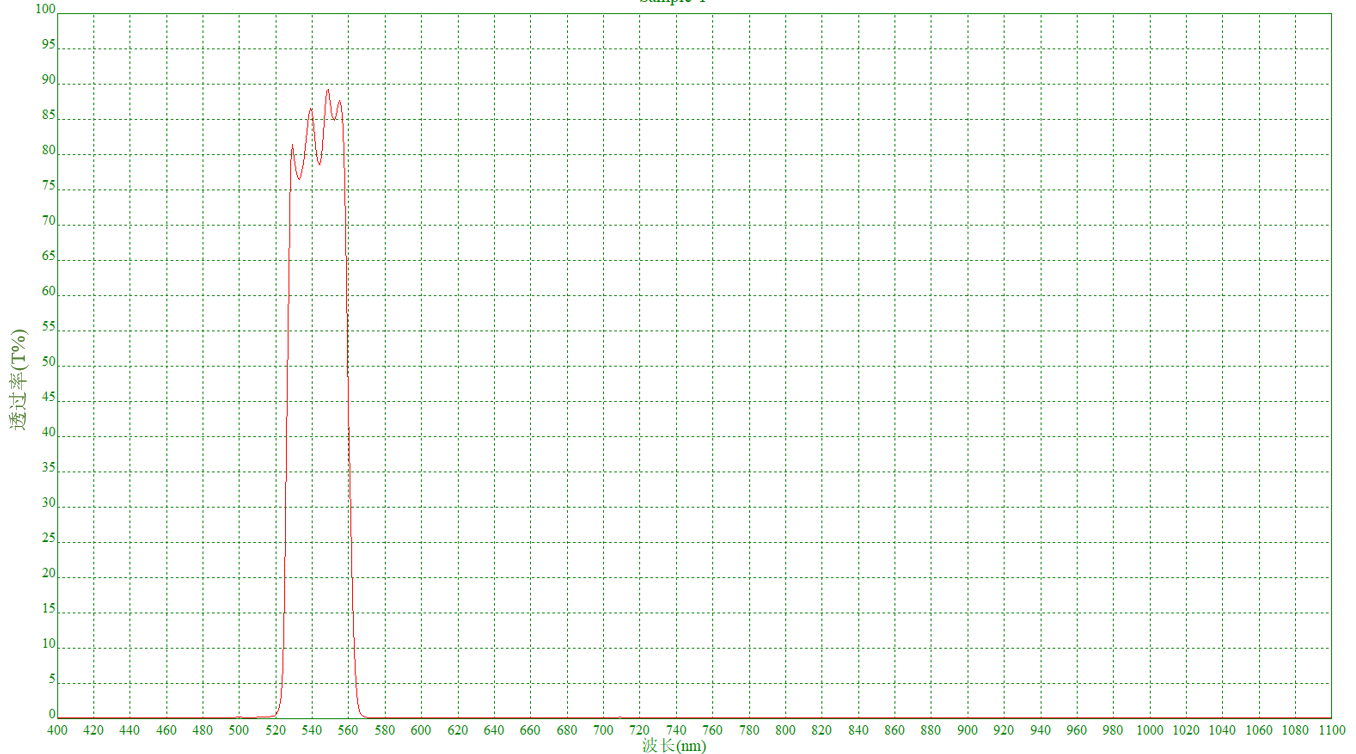


Figure S**22**. Transmittance spectra of band-pass optical filter BP570.


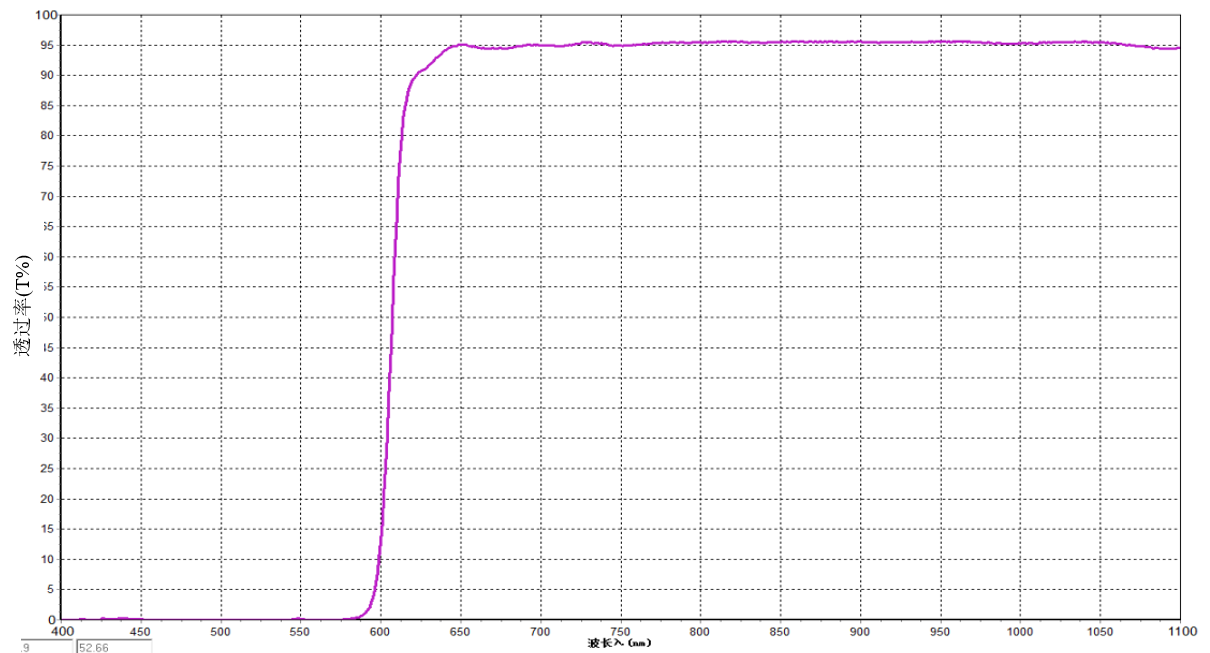


Figure S**23**. Transmittance spectra of long-pass optical filter LP605.


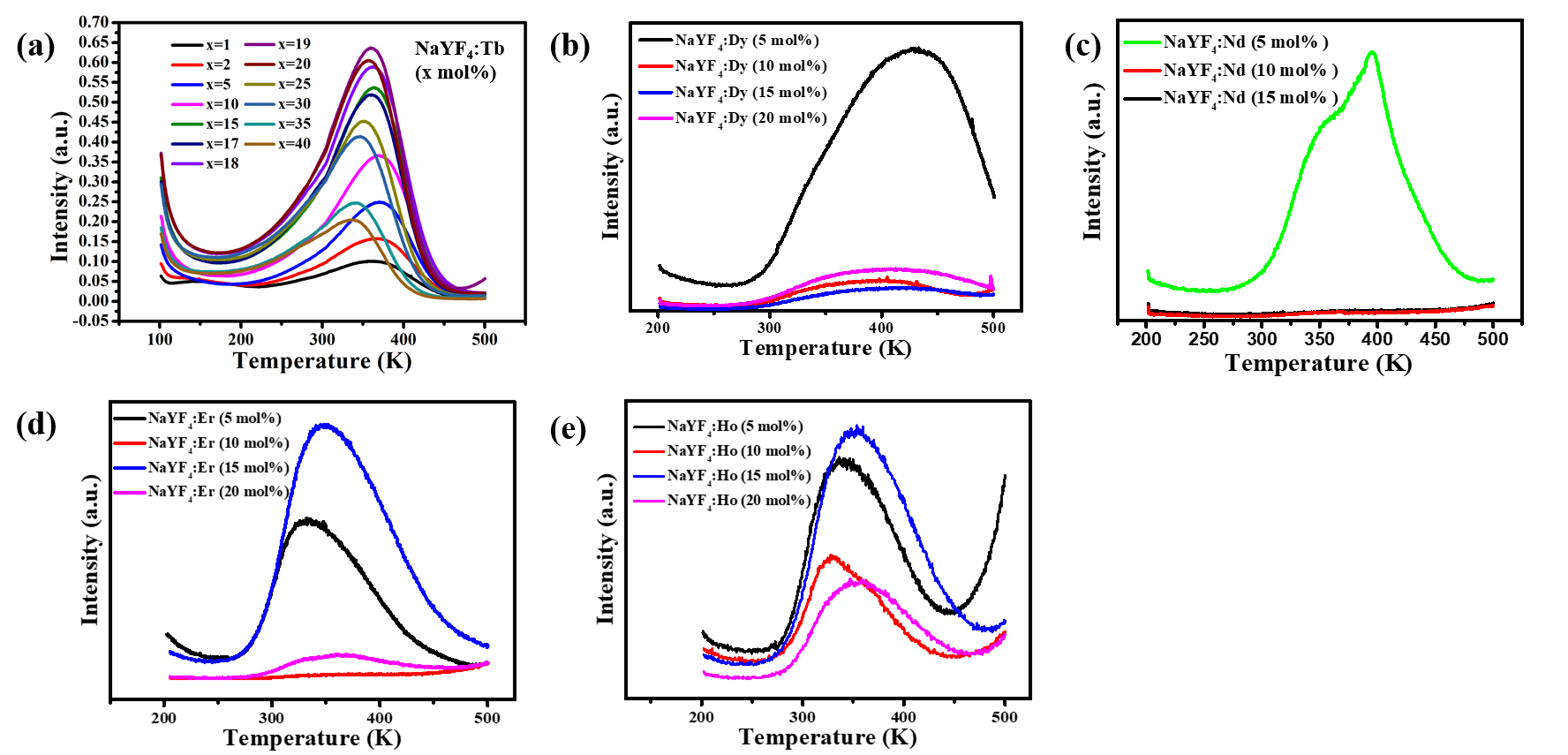


Figure S**24**. TL glow curves of the NaYF_4_:Tb^3+^(a), NaYF_4_:Dy^3+^ (b), NaYF_4_:Nd^3+^ (c), NaYF_4_:Er^3+^(d) and NaYF_4_:Ho^3+^ nanoparticles (e) with different doping concentrations. Generally, the different doping concentrations had little influence on the TL peak temperature while showed strong effects on the TL intensity. The nanoparticles giving the most intense TL intensity were considered as the ones with the optimal doping concentrations.


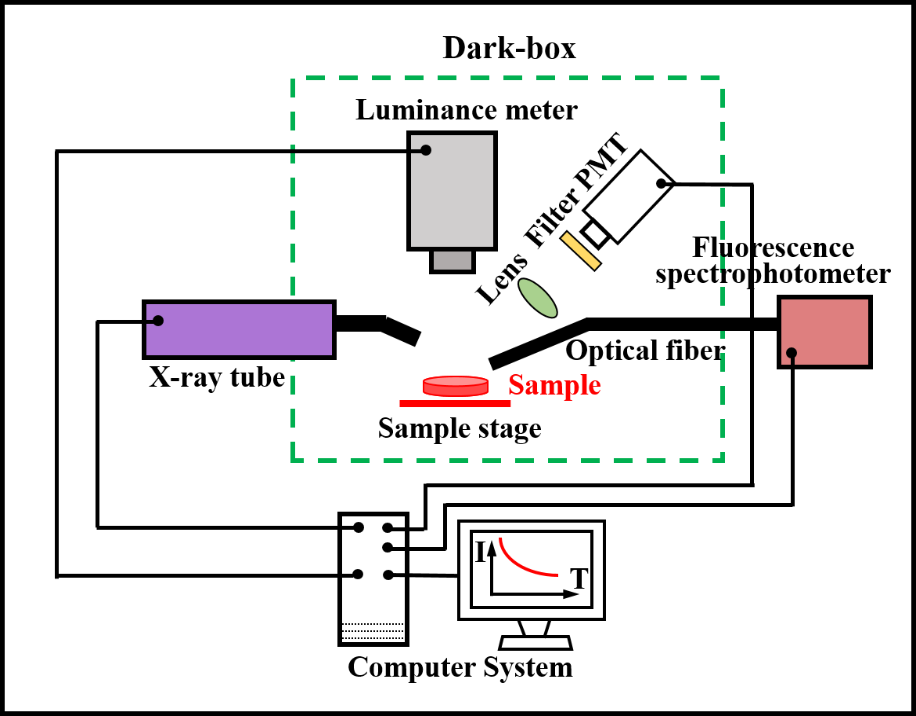


Figure S**25**. Schematic diagram of persistent luminescence (PersL) decay curve system. Excitation source came from an X-ray tube. A filter-attached photomultiplier tube (PMT), a fiber-type spectrophotometer and a luminance meter simultaneously monitored the PersL intensity.


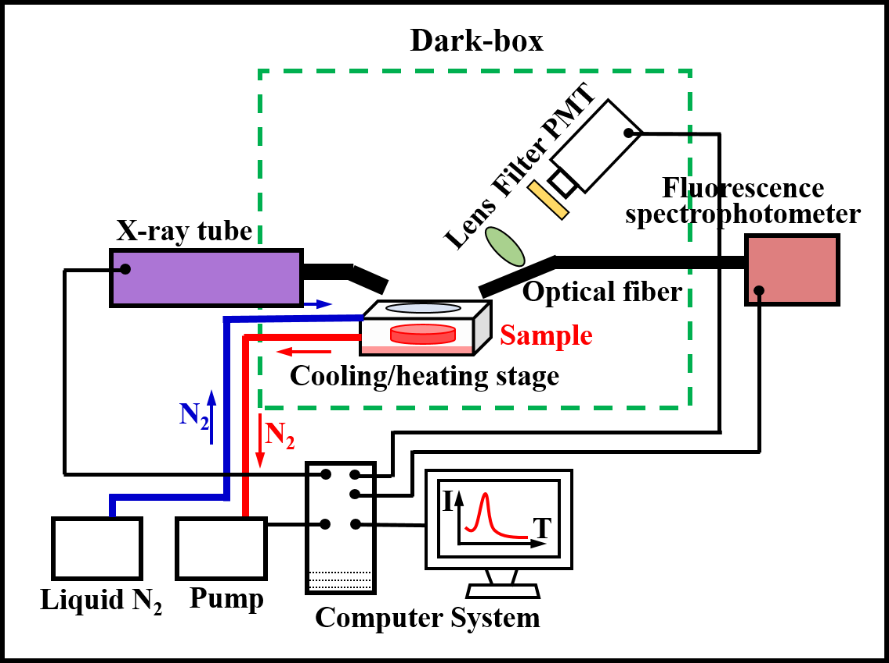


Figure S**26**. Schematic diagram of thermoluminescence (TL) glow curve measurement system. Excitation source came from an X-ray tube. The samples were placed inside a cooling/heating stage. A thin piece of quartz glass was installed on the top surface of the stage. Liquid N_2_ was pumped into the stage to cool sample, and gasified N_2_ was extracted out by a pump. The measurement system was real-time controlled by a LabVIEW-based customer-designed program.
